# NEIGHBORHOOD DISADVANTAGE AND PHYSICAL FUNCTIONING: EXPLORING GENDER-SPECIFIC MECHANISMS

**DOI:** 10.1093/geroni/igad104.3492

**Published:** 2023-12-21

**Authors:** Pui Yin Cheung, David Curtis, Ming Wen

**Affiliations:** University of Hong Kong, Hong Kong, Hong Kong; University of Utah, Salt Lake City, Utah, United States; University of Utah, Salt Lake City, Utah, United States

## Abstract

The relationship between poverty status and functional limitation among late middle age and older adults is well-established. However, the contextual effect of neighborhood disadvantage on physical functioning is less clear. First, the potential pathways linking the two are largely unexplored. Extant evidence shows that perceived neighborhood safety and order are significant predictors of physical functioning. Moreover, stress eating is a common coping strategy for stressful life events and contexts. These three explanations potentially mediate the relationship between poverty rate and physical functioning. Second, even though existing evidence finds stark gender difference in physical functioning, the explanations for the gender difference remains largely unknown. We propose that gender-specific mechanisms can explain the gender difference in physical functioning. The goal of the study was to test the gender-specific mechanism thesis based on a nationally representative sample of adults 55 to 93 years of age in the MIDUS 2 and 3 data (N = 2099). We employed Multilevel Generalized Structural Equation Modeling to test the gender-specific mediating roles of the three mediators. Results showed that county-level poverty rate is significantly associated with instrumental activities of daily living (IADL) among women and men. In contrast, perceived neighborhood safety and stress eating significantly mediate the relationship between poverty rate and physical functioning among women but not men. Furthermore, the indirect effect among women is significantly larger than among men. The important findings support gender-specific mechanisms linking neighborhood disadvantage and health and highlight the role of subjective neighborhood indicators among older women.

